# Predictive value of ellipsoid zone-related angle parameters in primary surgery of large macular hole: a case control study

**DOI:** 10.1186/s12886-023-03187-7

**Published:** 2023-11-17

**Authors:** Huaqin Xia, Jiarui Yang, Yi Ding, Xinglin Wang, Yanfeng Liao, Yiwen Shi, Hongliang Dou, Xuemin Li

**Affiliations:** 1 Department of Ophthalmology, Peking University Third Hospital, Beijing, China; 2 Beijing Key Laboratory of Restoration of Damaged Ocular Nerve, Peking University Third Hospital, Beijing, China; 3 Medical Oncology College, Beijing Shijitan Hospital, Capital Medical University, Beijing, China

**Keywords:** Macular hole, Ellipsoid zone, Vitrectomy

## Abstract

**Background:**

To explore the predictive value of Ellipsoid Zone (EZ) -related angle parameters for the outcome of primary macular hole surgery.

**Methods:**

This was a retrospective study. Patients diagnosed with large macular hole (MH) (minimum diameter > 500 μm) between 2018 and 2021 were enrolled. All patients underwent 25-gauge pars plana vitrectomy, internal limiting membrane (ILM) peeling and air tamponade. Spectral-domain OCT (SD-OCT) and best corrected visual acuity (BCVA) were measured in preoperative and 2-week post-operative. Classic and angle related parameters were measured via ImageJ. Angle regularity (AR) were defined as the standard deviation of the angle parameters in vertical and horizontal direction.

**Results:**

Seventy-six eyes were included for analysis; 24 eyes showed an unclosed macular hole at the 2-week postoperative and 52 eyes showed a closed hole. Preoperatively, MLD (*P* < 0.001), BD (*P* = 0.009) and diameter of EZ/ELM disruption (*P* = 0.002 and 0.025) in patients failed to close the hole after primary surgery were significantly larger than those succeeded. EZ-MH (*P* = 0.018), EZ-NFL (*P* = 0.006), EZ-GCL (*P* = 0.004), EZ-INL (*P* = 0.002), EZ-OPL (*P* = 0.009) and EZ-ONL (*P* = 0.011) angles were smaller in patients with unclosed hole. AR of the EZ-NFL (*P* = 0.009), EZ-GCL (*P* = 0.009), EZ-OPL (*P* = 0.023), EZ-ONL (*P* = 0.048) and Basal-NFL (*P* = 0.030) angles among the unclosed patients were significantly larger than those of the closed group. EZ-NFL (*P* = 0.015), EZ-GCL (*P* = 0.004), EZ-INL (*P* < 0.001), EZ-OPL (*P* < 0.001), EZ-ONL (*P* < 0.001), Basal (*P* = 0.023) and Basal-NFL (*P* < 0.001) angles of hole-unclosed patients enlarged significantly after the surgery.

**Conclusions:**

Patients with large macular holes and an increased EZ-related angle and angle AR are more likely to experience unsuccessful outcomes following primary MH surgery. Therefore, EZ-related angles hold potential as valuable parameters for predicting the surgical outcome.

**Supplementary Information:**

The online version contains supplementary material available at 10.1186/s12886-023-03187-7.

## Background

An idiopathic full-thickness macular hole (IFTMH) is a term used to describe a defect in the retina at the fovea, which is responsible for central vision decline. The worldwide incidence of IFTMH is estimated to be 8 cases per 100,000 individuals per year [1]. Pars plana vitrectomy, along with limiting membrane peeling and intravitreal gas tamponade, has been shown to significantly improve visual outcomes. Success rates for the closure of macular holes ranging from 85 to 100% in patients were achieved [2–4]. However, certain complex cases of macular holes have a higher risk of failure following primary surgery. It is well-known that patients with a longer duration of the disease, larger macular holes (with a minimum diameter of > 500 μm), and high myopia tend to be less responsive to primary surgery [5]. Among these factors, the size of the hole is particularly critical and associated with a lower closure rate [6]. Therefore, accurately estimating the risks of unsuccessful closure for large macular holes before surgery is crucial in determining the appropriate treatment strategy.

Currently, the ellipsoid zone (EZ) is considered to play a crucial role in the recovery of visual acuity following macular hole (MH) surgery [7–9], and successful closure of the MH is often attributed to the restoration of the EZ. In our previous study, we found a close association between EZ-related parameters (such as diameter of EZ disruption, thickness, reflectivity, and integrity of the EZ) and visual function outcomes [10]. Although we attempted to identify a correlation between preoperative EZ-related angle parameters and postoperative visual outcomes, no significant findings were observed. However, the predictive value of EZ-related angles in the context of larger MHs remains unclear, and additional publications on this topic are currently lacking. Considering the close relationship between the EZ and functional recovery, we conducted further investigation into the role of EZ-related angle parameters in the closure of large MHs.

The aim of this study was to explore the predictive value of EZ-related angle parameters for the outcome of primary surgery and investigate how angle parameters change after unsuccessful surgery among patients with large MHs.

## Materials and methods

### Patient

This retrospective study was conducted in accordance with the ethical guidelines set forth by the Institutional Review Board of Peking University Third Hospital and complied with the principles outlined in the Declaration of Helsinki (Ethical approval number: IRB00006761-M2022345). Informed consent was obtained from all participating subjects. The reporting of our study adhered to the TRIPOD (Transparent Reporting of a multivariable prediction model for Individual Prognosis or Diagnosis) checklist [11]. Subjects who met the following criteria were enrolled in this study: diagnosed with large MH between 2018 and 2021 with following up after surgery; spectral-domain OCT (SD-OCT) scans at the preoperative visit and 2-week post-operative visit; minimum diameter of MH was larger than 500 μm. A closure of macular hole was considered if there was complete circumferential hole rim attachment [9].

**Fig. 1:**
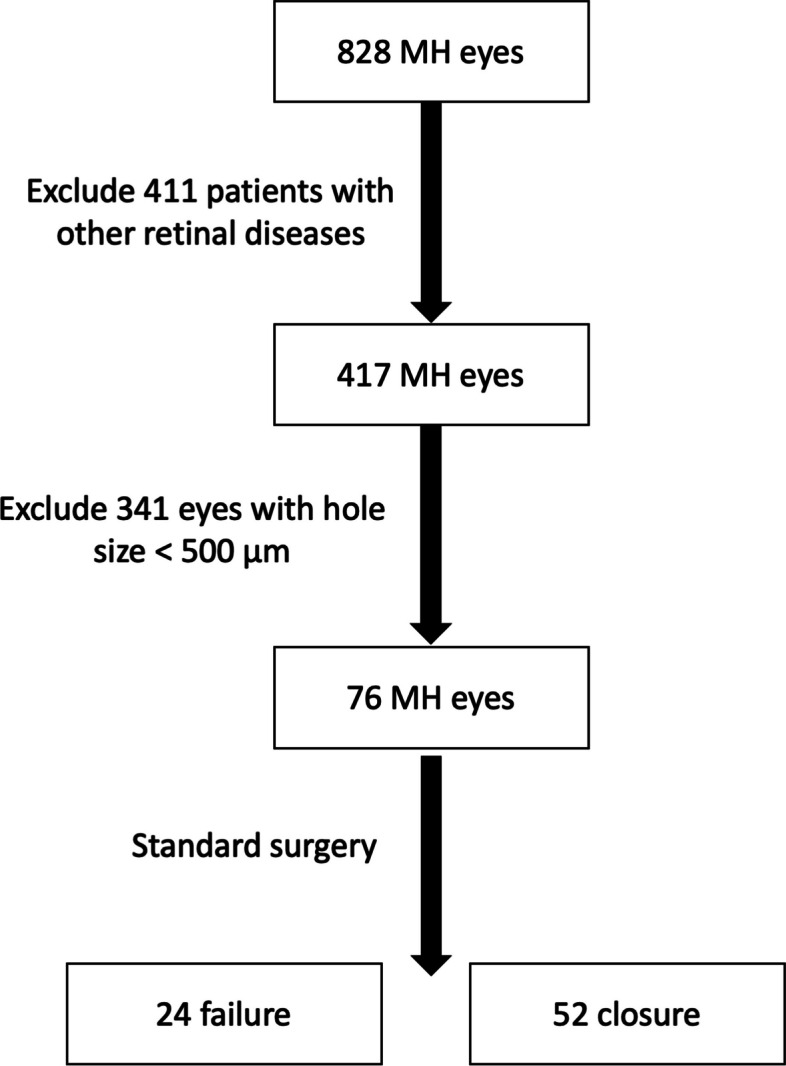
Flow chart of case selection

We excluded patients with an axial length (AL) greater than 26 mm, as well as those with other retinal diseases such as age-related macular degeneration or diabetic retinopathy. Patients who had previously undergone retina surgery or those with incomplete medical records were also excluded (Fig. 1).

Demographic information was obtained from the medical records, and the axial length was measured using the IOL Master 700 (Carl Zeiss Meditec, Jena, Germany). Best corrected visual acuity (BCVA) was measured using a standard logarithmic visual acuity chart and was converted to logMAR units for statistical analysis.

All patients underwent 25-gauge pars plana vitrectomy combining with cataract phacoemulsification and intraocular lens implantation if there was lens opacity. Indocyanine green (0.25%) diluted in 10% glucose was used to assist ILM peeling with peeling radius of 2 disc diameter, finally complete fluid-air exchange was done. All patients were instructed to maintain a prone position for at least 3 days.

### OCT

**Fig. 2:**
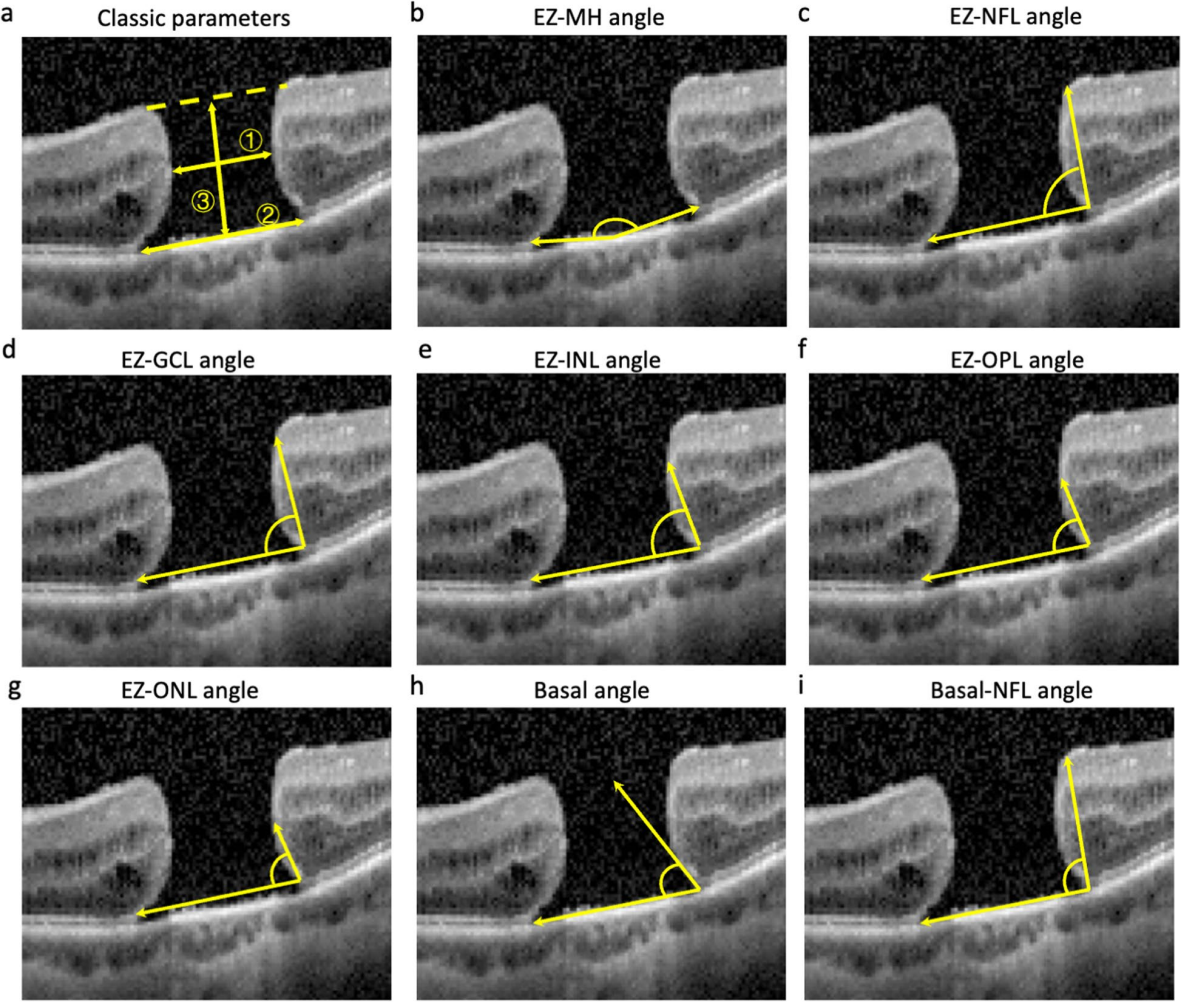
Diagrams showing the angles measured. **a** ① Minimal linear diameter, MLD; ② Basal diameter, BD; ③ Height, H; (**b**) EZ-MH angle: the angle whose vertex was located at the center of the hole, and the endpoints of both sides were located at the anterior border of the EZ band edge, (**c**) EZ-NFL angle, (**d**) EZ-GCL angle, (**e**) EZ-INL angle, (**f**) EZ-OPL angle, (**g**) EZ-ONL angle. The EZ-NFL, EZ-GCL, EZ-INL, EZ-OPL, and EZ-ONL angles were each between a line connecting the two upper edges of the EZ band and a line through the upper edge of the respective band. **h** Basal angle formed by the base of the macular hole and the RPE layer, (**i**) Basal-NFL angle between a line connecting the base of the macular hole and the edge of the NFL and the line of the RPE

OCT images were captured using SD-OCT in Autoscanning mode (Spectralis; Heidelberg Engineering, Heidelberg, Germany). According to previous study, holes in 94.6% of patients with large macular hole were closed within 1 week after surgery [12]. Therefore, we perform SD-OCT scan before the surgery and at 2 weeks after surgery. The scale of the horizontal and vertical bar is four to one. The minimum linear diameter (MLD), basal diameter (BD), height (H) of MH, diameter of ELM disruption, diameter of EZ disruption and EZ-related angle parameters were measured using ImageJ software (1.47v, Wayne Rasband, National Institutes of Health, Bethesda, MD, USA, http://imagej.nih.gov/ij) on the horizontal and vertical orientation of the OCT image (Fig. 2a). The EZ-related angle parameters included the EZ-MH angle, EZ-nerve fiber layer (EZ-NFL) angle, EZ-ganglion cell layer (EZ-GCL) angle, EZ-internal nuclear layer (EZ-INL) angle, EZ-outer plexiform layer (EZ-OPL) angle, EZ-outer nuclear layer (EZ-ONL) angle, Basal angle and Basal-NFL angle [10]. Unless otherwise stated, the EZ-MH angle, diameter of the EZ and ELM disruption in the following paragraph represent the average of the two measurements in the horizontal and vertical scans, and the other EZ-related angle parameters represent the average of the four measurements in the nasal, temporal, superior and inferior scans. The EZ-MH angle referred to the angle whose vertex was located at the center of the hole, and the endpoints of both sides were located at the anterior border of the EZ band edge. The EZ-NFL, EZ-GCL, EZ-INL, EZ-OPL, and EZ-ONL angles were considered the intersection angle between a line connecting the two upper edges of the EZ band and a line through the upper edge of the respective band. The basal angle refers to the angle formed by the base of the macular hole and the retinal pigment epithelium (RPE) layer, whereas the basal-NFL angle was between a line connecting the base of the macular hole and the edge of the NFL and the line of the RPE (Fig. 2 b-i). In addition, we put forth a new parameter called the angle regularity (AR) referring to the standard deviation of the angle parameters in four directions. Other indexes were calculated from the above parameters: diameter hole index (DHI) = MLD/BD, MH index (MHI) = H/BD, and traction hole index (THI) = H/MLD. All measurements were performed twice by HX, and the average of the two measurements was used for analysis.

### Statistical analysis

All statistical analyses were performed using IBM SPSS for Mac version 26.0 (IBM Corp., Armonk, NY, USA). The Mann‒Whitney U test was used to compare angle parameters between closed and unclosed patients, and the Wilcoxon signed-rank test was used to compare the difference in preoperative and postoperative angle parameters among unclosed patients. To compare classic preoperative parameters (such as MLD, BD, H, diameter of EZ and ELM disruption) and other indexes (including DHI, MHI, and THI), independent t-tests were performed if they exhibited homogeneity of variance according to Levene’s test. If the data did not meet the assumptions of normality or homogeneity of variance, nonparametric analysis (such as the Mann–Whitney U test) was utilized. Correlation analysis was used to explore correlations between classic preoperative parameters and preoperative angle parameters. *P* < 0.05 was considered statistically significant.

## Results

### Basic characteristics and classic parameters

Seventy-six eyes (36 OD, 40 OS) of 75 patients receiving PPV were included for analysis. Among them, 24 eyes (11 OD, 13 OS) of 24 patients (32%) (3 male, 21 female) showed an unclosed macular hole at the 2-week postoperative visit, and 52 eyes (25 OD, 27 OS) of 51 patients (68%) (10 male, 41 female) showed a closed macular hole at the 2-week postoperative visit (Table 1). MLD (*P* < 0.001), BD (*P* = 0.009) and diameter of EZ/ELM disruption (*P* = 0.002/0.025) in the unclosed group were significantly larger than those in the closed group.

**Table 1:** Basic characteristics and classic parameters

Item	Unclosed (n = 24)	Closed (n = 52)	*P*
Age, years	66.50 ± 8.75	61.73 ± 11.31	0.072
BCVA, log MAR	1.22 ± 0.35	1.19 ± 0.32	0.699
AL, mm	23.68	23.84	0.681
MLD, μm	786.91 ± 202.33	612.06 ± 152.72	< 0.001***
BD, μm	1488.51 ± 457.12	1237.02 ± 321.08	0.009**
H, μm	381.94 ± 117.70	342.12 ± 82.48	0.105
EZ disruption, μm	1978.19 ± 542.48	1602.05 ± 424.99	0.002**
ELM disruption, μm	1818.98 ± 602.58	1484.19 ± 367.48	0.025*
DHI	0.55 ± 0.12	0.51 ± 0.09	0.121
MHI	0.27 ± 0.11	0.29 ± 0.08	0.424
THI	0.54 ± 0.29	0.58 ± 0.22	0.506
CME (yes/no)	21/3	45/7	0.610

All values are the mean ± standard deviation unless otherwise indicated

*BCVA* best corrected visual acuity, *MLD* minimal linear diameter, *BD* basal diameter, *H* height, *EZ* ellipsoid zone, *ELM* external limiting membrane, *DHI* diameter hole index, *MHI* macular hole index, *THI* traction hole index

*P* < 0.05 is marked with *, *P* < 0.01 is marked with ** and *P* < 0.001 is marked with ***

### Preoperative parameters of closure and non-closure MH

The EZ-MH angle demonstrated a significant difference between the closed and unclosed MH groups (p = 0.018). In addition, the EZ-NFL, EZ-GCL, EZ-INL, EZ-OPL and EZ-ONL angles were smaller in the unclosed group (*P* = 0.006, *P* = 0.004,* P* = 0.002, *P* = 0.009 and* P* = 0.011, respectively). However, the basal and mean basal-NFL angles showed no statistical significance (*P* = 0.465 and *P* = 0.940, respectively) (Table 2).

**Table 2:** Preoperative parameter comparisons between closed and unclosed MHs

Item	Unclosed MH	Closed MH	*P*
EZ-MH angle	170.14 ± 3.70	167.62 ± 5.49	0.018*
EZ-NFL angle	75.39 ± 6.73	79.91 ± 7.45	0.006**
EZ-GCL angle	71.98 ± 6.86	76.81 ± 7.74	0.004**
EZ-INL angle	63.54 ± 8.51	69.82 ± 8.56	0.002**
EZ-OPL angle	57.93 ± 11.56	64.82 ± 9.66	0.009**
EZ-ONL angle	53.97 ± 12.97	61.64 ± 10.44	0.011*
Basal angle	115.30 ± 6.98	116.43 ± 7.33	0.465
Basal-NFL angle	91.13 ± 6.07	91.68 ± 5.97	0.940

All values are the mean ± standard deviation unless otherwise indicated

*MH* macular hole, *EZ* ellipsoid zone, *NFL* nerve fiber layer, *GCL* ganglion cell layer, *INL* inner nuclear layer, *ONL* outer nuclear layer, *OPL* outer plexiform layer

*P* < 0.05 is marked with *, *P* < 0.01 is marked with ** and *P* < 0.001 is marked with ***

**Fig. 3:**
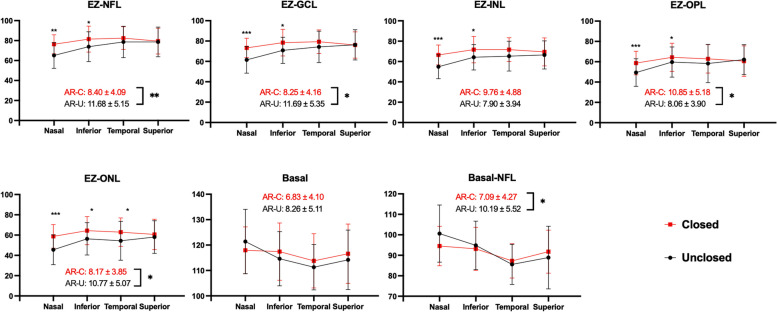
Comparison of angles from different directions and AR of each angle between the two groups. EZ, ellipsoid zone; NFL, nerve fiber layer; GCL, ganglion cell layer; INL, inner nuclear layer; ONL, outer nuclear layer; OPL, outer plexiform layer; AR, angle regularity. *P* < 0.05 is marked with *, *P* < 0.01 is marked with ** and *P* < 0.001 is marked with ***

For each direction, the significant differences between closed and unclosed MH in nasal direction of EZ-NFL, EZ-GCL, EZ-INL, EZ-OPL and EZ-ONL (*P* = 0.001, *P* < 0.001,* P* < 0.001, *P* < 0.001 and *P* ≤ 0.001, respectively), temporal direction of EZ-ONL (*P* = 0.044) and inferior direction of EZ-NFL, EZ-GCL, EZ-OPL, EZ-ONL and Basal-NFL angle (*P* = 0.009, *P* = 0.009,* P* = 0.023, *P* = 0.048 and *P* = 0.030, respectively) were found. (Fig. 3, Supplementary Table 1). The ARs of the EZ-NFL, EZ-GCL, EZ-OPL, EZ-ONL and Basal-NFL angles in the unclosed group were significantly larger than those of the closed group (*P* = 0.009, *P* = 0.009, *P* = 0.023, *P* = 0.048 and *P* = 0.030, respectively), whereas the other 2 parameters (EZ-INL and Basal angle) showed no difference (*P* = 0.145 and *P* = 0.223) between them.

### Correlation between preoperative EZ-related angle parameters and classic OCT parameters

Correlation analysis indicated that MLD was negatively correlated with the EZ-GCL, EZ-INL, EZ-OPL and EZ-ONL angles (*P* = 0.025, *P* < 0.001, *P* < 0.001 and *P* ≤ 0.001, respectively), while BD did not significantly correlate with the EZ-related angles, instead, it positively correlated with the Basal (*P* ≤ 0.001) and Basal-NFL (*P* ≤ 0.001) angles (Table 3). Regarding the diameter of the EZ and ELM disruption, all seven angle parameters had significantly negative correlation with the EZ-related angles and a positive correlation with the Basal and Basal-NFL angles. Notably, the correlation coefficient was higher for the EZ-related angle in the upper layer of the retina, suggesting a stronger association between the upper layer EZ-related angle and the diameter of EZ disruption.

**Table 3:** Correlation between EZ-related angle parameters and classic OCT parameters

	MLD		BD		Diameter of EZ disruption		Diameter of ELM disruption	
	CC	*P*	CC	*P*	CC	*P*	CC	*P*
EZ-NFL	-0.204	0.086	-0.400	0.001**	-0.748	< 0.001***	-0.450	< 0.001***
EZ-GCL	-0.264	0.025*	-0.411	< 0.001***	-0.738	< 0.001***	-0.474	< 0.001***
EZ-INL	-0.418	< 0.001***	-0.460	< 0.001***	-0.709	< 0.001***	-0.527	< 0.001***
EZ-OPL	-0.420	< 0.001***	-0.418	< 0.001***	-0.645	< 0.001***	-0.516	< 0.001***
EZ-ONL	-0.444	< 0.001***	-0.417	< 0.001***	-0.630	< 0.001***	-0.509	< 0.001***
Basal	0.168	0.156	0.511	< 0.001***	0.356	0.002**	0.430	< 0.001***
Basal-NFL	0.073	0.545	0.526	< 0.001***	0.385	0.001**	0.397	0.001**

*MLD* minimal linear diameter, *BD* basal diameter, *EZ* ellipsoid zone, *ELM* external limiting membrane, *CC* correlation coefficient, *NFL* nerve fiber layer, *GCL* ganglion cell layer, *INL* inner nuclear layer, *ONL* outer nuclear layer, *OPL* outer plexiform layer

*P* < 0.05 is marked with *, *P* < 0.01 is marked with ** and *P* < 0.001 is marked with ***

### Preoperative and postoperative parameters among unclosed MH patients

The mean BCVA (logMAR) of unclosed group did not significantly change from 1.22 ± 0.34 in preoperative to 1.22 ± 0.31 in postoperative (*P* = 0.943). Meanwhile, the mean BCVA (logMAR) of closed group somewhat improved from 1.19 ± 0.32 in baseline to 1.07 ± 0.60 in last follow-up (*P* = 0.180). The significant changes in preoperative and postoperative were observed in other three parameters, namely the mean MLD 662.36 ± 185.05 μm vs 559.23 ± 204.71 μm, BD 1309.37 ± 379.75 μm vs 868.34 ± 316.69 μm and H 353.57 ± 94.85 μm vs 240.15 ± 69.03 μm in the unclosed group (*P* = 0.001, *P* < 0.001, *P* < 0.001, respectively). Nonetheless, there is no significance found in the mean diameter of EZ and ELM disruption (Supplementary Table 1). As for angle parameters, no difference was found in the EZ-MH (*P* = 0.741), while there were significant changes in the EZ-NFL, EZ-GCL, EZ-INL, EZ-OPL, EZ-ONL, Basal and Basal-NFL angles (*P* = 0.015, *P* = 0.004, *P* < 0.001, *P* < 0.001, *P* < 0.001, *P* = 0.023 and *P* < 0.001, respectively) (Supplementary Table 2). Comparing with baseline, the EZ-NFL (*P* = 0.001 and *P* = 0.019), EZ-GCL (*P* < 0.001 and *P* = 0.024), EZ-INL (*P* < 0.001 and *P* = 0.010), EZ-OPL (*P* < 0.001 and *P* = 0.037) and EZ-ONL (*P* < 0.001 and *P* = 0.037) in nasal and inferior direction became smaller. Similar change of EZ-ONL (*P* = 0.044) in temporal direction, MHI and THI in preoperative and postoperative showed no significant difference (*P* = 0.131 and *P* = 0.689), but the DHI were found to be different (*P* = 0.013).

For the AR of each angle, only the AR of the basal angle displayed a significant difference (*P* = 0.005), whereas the EZ-NFL, EZ-GCL, EZ-INL, EZ-OPL, EZ-ONL and basal-NFL angles showed no significant difference (*P* = 0.737, *P* = 0.823, *P* = 0.117, *P* = 0.478, *P* = 0.478 and *P* = 0.263, respectively).

## Discussion

Based on our clinical observations, the closure rate of large macular holes remains unsatisfactory. Given the significant role of EZ recovery in visual improvement, it is possible that EZ-related parameters have the potential to predict the early outcome of surgery. However, we found a lack of studies examining the relationship between EZ-related parameters and the early evaluation of the success of primary macular hole surgery. In our previous study, we discovered that the recovery of the EZ itself, rather than EZ-related angles, was significantly associated with postoperative visual acuity in patients with successfully closed macular holes [10]. In this study, we aimed to further explore the predictive value of EZ-related angle parameters in the successful closure of large macular holes. Our results revealed that preoperative EZ-NFL, EZ-GCL, EZ-INL, EZ-OPL, and EZ-ONL angles were significantly larger in patients with closed macular holes compared to those with unclosed macular holes after surgery. Additionally, we introduced a new parameter called angle regularity, which represented the degree of irregularity in the aforementioned angles across four directions. It was observed that the AR of unclosed patients was significantly larger than that of closed patients, particularly in the nasal direction. This may suggest varying degrees of retinal damage in the nasal, inferior, temporal, and superior directions. Comparison of preoperative and postoperative parameters in unclosed patients revealed some degree of recovery in structure-related parameters, despite the lack of significant improvement in postoperative visual acuity.

Our findings indicate that the MLD, BD, diameter of ELM and EZ disruption were significantly larger in patients with unclosed macular holes compared to those with closed holes. However, no significant difference was observed for H. These results align with previous studies that have demonstrated the predictive value of BD as the strongest indicator for anatomical and functional prognosis in patients after macular hole surgery [8, 9, 13]. Other studies demonstrated that the angle between the upward edge of the retinal surface and RPE did not significantly correlate with the type of MH closure [14], our research also showed no significant difference between the two groups of patients before the surgery. Thus, further investigation is needed to identify more valuable angle parameters that accurately predict surgical outcomes.

In our study, we introduced EZ-related angle parameters to explore their predictive value for successful macular hole (MH) surgery. We found that the EZ-NFL, EZ-GCL, EZ-INL, EZ-OPL, and EZ-ONL angles in patients with closed MHs were significantly larger than those in patients with unclosed MHs before surgery. Since H was comparable between the two groups, the size of the EZ-related angle was largely dependent on the diameter of EZ disruption. Because unclosed MH patients tended to have a larger size of EZ disruption, the EZ-related angles in the unclosed group were significantly larger than those in the closed group. These findings indicated that only EZ-related angles, rather than classic parameters related angle (Basal or Basal-NFL angles), were capable of predicting prognosis. We propose that classical parameters such as MLD, BD, and the diameter of EZ disruption are one-dimensional parameters that reflect length, while angles are two-dimensional parameters that encompass these length measurements. Therefore, angles can provide a more comprehensive description of the anatomical status of the macula compared to length parameters alone. Consequently, EZ-related angle parameters may be more accurate in predicting the outcome of primary MH surgery compared to classical length parameters. We propose that classical parameters such as MLD, BD, and the diameter of EZ disruption are one-dimensional parameters that reflect length, while angles are two-dimensional parameters that encompass these length measurements. Therefore, angles can provide a more comprehensive description of the anatomical status of the macula compared to length parameters alone. Consequently, EZ-related angle parameters may be more accurate in predicting the outcome of primary MH surgery compared to classical length parameters. Importantly, the EZ represents the mitochondria-rich region of the photoreceptors [15], and it reflects the functional status of these photoreceptors. Therefore, damage to the EZ, comparing with other structures, was more indicative of the extent of macular function affected by the hole. EZ disruption appears to be a more valid predictor of functional recovery after surgery, which may explain why the basal angle or basal-NFL angle alone is not able to predict the prognosis of a macular hole.

In our study, we introduced the concept of angle regularity (AR) to assess the uniformity of angle distribution in closed and unclosed macular holes (MHs), which may have an impact on the prognosis of MH surgery. Our findings revealed that the AR of most angles in unclosed patients was significantly higher compared to closed patients, indicating that the distribution pattern of angles could affect the success rate of surgery in patients with large MHs. Furthermore, we observed that the main differences in angles between the two groups were primarily in the nasal and inferior directions, with the nasal direction showing the most significant disparity. It is widely accepted that posterior vitreous detachment (PVD) plays a critical role in the formation of MHs [16]. During the early stages of PVD, the perifoveal vitreous detaches, while the vitreofoveal adhesion and optic disc adhesion persist [17]. Subsequently, the vitreofoveal vitreous detaches before the optic disc, leading to traction forces that contribute to the formation of a macular hole. Based on our speculation, during this stage, the vitreous traction on the retina mainly remains at the optic disc, resulting in a persistent traction on the nasal side of the macula. This may explain the more significant differences observed in the angles in the nasal direction. Additionally, the papillomacular bundle, which contains a higher fiber density and shorter nerve fibers compared to other regions, is located on the nasal side of the macula [18]. This region presumably produces greater tension than in other directions and more likely results in significant differences. Therefore, optimizing surgical techniques to release nasal traction as much as possible may increase the likelihood of successful closure of the macular hole.

In our study, we conducted a comparison of parameters before and after surgery in patients with unclosed macular holes. The results revealed that after surgery, the MLD, BD and H of the MH significantly decreased. However, there was no statistical significance observed in the diameter of the EZ and ELM. Previous studies by Ikuno et al. [19] and John et al. [20] reported an increase in the size of the macular hole after unsuccessful surgery. They noted an enlargement of the hole from 480 μm to 610 μm and from 594 μm to 675 μm, respectively. However, our study demonstrated a dramatic reduction in the size of the hole after surgery. This difference could be attributed to variations in the follow-up period, as the previous studies had a minimum follow-up period of three months, while our study focused on early postoperative changes. It is possible that the hole may become larger over time in patients who did not achieve successful closure in the primary surgery. Consistent with previous findings, our study also indicated that unclosed patients did not experience significant functional improvement in BCVA [20]. We observed that almost all EZ-related angles significantly decreased after surgery. This could be attributed to the peeling of the ILM, as the release of macular traction allowed each retinal layer to return to its normal position. Additionally, we noticed the alleviation of cystoid macular edema (CME), which facilitated the recovery of angle parameters and contributed to the relief of extra tension on the macula. Our results demonstrate that surgery can release the traction on the macula, prevent the enlargement of the macular hole, and allow each retinal layer to return to its normal position, even in cases where the closure of the MH was not achieved.

Our study had some limitations. Firstly, we only analyzed the short-term risk factors of patients with large MHs. To fully understand the long-term risk factors, a more extended follow-up duration is necessary. Secondly, our sample size was small, as it was limited to matching patients with similar characteristics in terms of BD, MLD and diameter of EZ disruption. Therefore, subsequent studies are needed to expand the sample size to evaluate the predictive value of the angle parameters when matching these classical parameters. Furthermore, our study only included patients with stage IV macular holes. However, we found that the diameter of EZ disruption was significantly longer than the size of the hole itself, so further study may construct the model of EZ damage in patients with different stages of MH and modify grading system combining EZ parameters to reflect functional damage of MH patients. This would provide valuable evidence for determining the optimal timing and appropriate surgical intervention procedures. Due to the nature of retrospective studies, patients all underwent standard ILM peeling without modified procedure, and in the future, we will enroll patients with modified ILM peeling techniques to gain a better prognosis.

In conclusion, our findings support the notion that large macular holes, characterized by large EZ-related angle and angle AR, are more likely to have unfavorable outcomes following primary MH surgery. The EZ-related angles demonstrate potential as valuable parameters in predicting the prognosis of surgical interventions. Notably, the angles in the nasal direction were found to be particularly influential. Consequently, it is crucial to release nasal traction during surgery to improve the likelihood of a favorable prognosis.

### Supplementary Information

**Additional file 1: Supplementary Table 1.** Comparison of angles from different directions and AR of each angle between the two groups.

**Additional file 2: Supplementary Table 2.** Classic preoperative and postoperative parameter comparisons among unclosed MH patients

**Additional file 3: Supplementary Table 3.** EZ-related preoperative and postoperative parameter comparisons among unclosed MH patients

## Data Availability

The datasets used and analyzed during the current study are available from the corresponding author on reasonable request.

## Abbreviations

EZ: Ellipsoid Zone
AL: Axial Length
MH: Macular Hole
AR: Angle Regularity
MLD: Minimum Linear Distance
BD: Basal Distance
ELM: External Limiting Membrane
OCT: Optical Coherence Tomography
ILM: Internal Limiting Membrane
BCVA: Best Corrected Visual Acuity
NFL: Nerve Fiber Layer
GCL: Ganglion Cell Layer
INL: Internal Nuclear Layer
OPL: Outer Plexiform Layer
ONL: Outer Nuclear Layer
RPE: Retinal Pigment Epithelium
