# Supplementary material for: Predictive value of ellipsoid zone-related angle parameters in primary surgery of large macular hole: a case control study
Source: BMC Ophthalmol. 2023 Nov 17;23:467. doi: 10.1186/s12886-023-03187-7 (PMC10655441; doi:10.1186/s12886-023-03187-7)
Supplement: Supplementary file 1 — Additional file 1: Supplementary Table 1. Comparison of angles from different directions and AR of each angle between the two groups. [file 12886_2023_3187_MOESM1_ESM.docx]

Supplementary Table 1 Comparison of angles from different directions and AR of each angle between the two groups.

|  | Unclosed（n=24） | | | | |  | Closed (n=52) | | | | |  | *P* value | | | | |
| --- | --- | --- | --- | --- | --- | --- | --- | --- | --- | --- | --- | --- | --- | --- | --- | --- | --- |
|  | Nasal | Temporal | Superior | Inferior | AR |  | Nasal | Temporal | Superior | Inferior | AR |  | Nasal | Temporal | Superior | Inferior | AR |
| EZ-NFL | 65.13 ± 12.92 | 78.59 ±15.73 | 78.60 ± 14.88 | 73.87 ± 13.03 | 11.68 ± 5.15 |  | 76.30 ± 9.49 | 82.42 ± 11.36 | 79.46 ± 12.89 | 81.47 ± 12.99 | 8.40 ± 4.09 |  | 0.001** | 0.325 | 0.836 | 0.019* | 0.009** |
| EZ-GCL | 61.55 ± 13.08 | 74.31 ± 15.45 | 76.29 ± 14.81 | 70.88 ± 12.85 | 11.69 ± 5.35 |  | 73.31 ± 9.55 | 79.37 ± 11.58 | 76.09 ± 12.91 | 78.46 ± 13.10 | 8.25 ± 4.16 |  | < 0.001*** | 0.191 | 0.951 | 0.024* | 0.009* |
| EZ-INL | 54.89 ± 11.73 | 65.30 ± 14.71 | 66.48 ± 13.82 | 64.20 ± 12.52 | 9.76 ± 4.88 |  | 66.48 ± 9.80 | 71.69 ± 11.68 | 69.43 ± 13.75 | 71.70 ± 13.01 | 7.90 ± 3.94 |  | < 0.001*** | 0.053 | 0.180 | 0.010* | 0.145 |
| EZ-OPL | 49.34 ± 13.63 | 58.30 ± 18.81 | 62.16 ± 14.80 | 59.67 ± 14.85 | 10.85 ± 5.18 |  | 61.78 ± 11.17 | 66.24 ± 13.20 | 64.00 ± 14.29 | 67.26 ± 13.34 | 8.06 ± 3.90 |  | < 0.001*** | 0.057 | 0.407 | 0.037* | 0.023* |
| EZ-ONL | 45.55 ± 14.70 | 54.36 ± 19.18 | 58.03 ± 16.08 | 56.26 ± 15.93 | 10.77 ± 5.07 |  | 58.67 ± 11.63 | 62.89 ± 14.01 | 60.60 ± 14.99 | 64.41 ± 13.85 | 8.17 ± 3.85 |  | < 0.001*** | 0.044* | 0.309 | 0.037* | 0.048* |
| Basal | 121.38 ± 12.68 | 111.27 ± 8.95 | 114.17 ± 11.71 | 114.61 ± 10.68 | 8.26 ± 5.11 |  | 117.94 ± 9.21 | 113.80 ± 10.68 | 116.57 ± 11.67 | 117.41 ± 11.28 | 6.83 ± 4.10 |  | 0.441 | 0.309 | 0.443 | 0.237 | 0.223 |
| Basal-NFL | 100.61 ± 13.96 | 85.55 ± 9.80 | 88.89 ± 15.31 | 94.86 ± 11.85 | 10.19 ± 5.52 |  | 94.53 ± 9.65 | 87.29 ± 8.40 | 91.75 ± 10.81 | 93.15 ± 10.50 | 7.09 ± 4.27 |  | 0.069 | 0.546 | 0.567 | 0.821 | 0.030* |

*All values are the mean ± standard deviation unless otherwise indicated. EZ, ellipsoid zone; AR, angle regularity; NFL, nerve fiber layer; GCL, ganglion cell layer; INL, inner nuclear layer; OPL, outer plexiform layer; ONL, outer nuclear layer. P<0.05 is marked with *, P<0.01 is marked with ** and P<0.001 is marked with ***.*
