# Supplementary material for: Predictive value of ellipsoid zone-related angle parameters in primary surgery of large macular hole: a case control study
Source: BMC Ophthalmol. 2023 Nov 17;23:467. doi: 10.1186/s12886-023-03187-7 (PMC10655441; doi:10.1186/s12886-023-03187-7)
Supplement: Supplementary file 2 — Additional file 2: Supplementary Table 2. Classic preoperative and postoperative parameter comparisons among unclosed MH patients [file 12886_2023_3187_MOESM2_ESM.docx]

Supplementary Table 2 Classic preoperative and postoperative parameter comparisons among unclosed MH patients

| Item | Preop | Post-op | *P* |
| --- | --- | --- | --- |
| BCVA, log MAR | 1.22 ± 0.35 | 1.22 ± 0.31 | 0.943 |
| Mean MLD, μm | 662.36 ± 185.05 | 559.23 ± 204.71 | 0.001** |
| Mean BD, μm | 1309.37 ± 379.75 | 868.34 ± 316.69 | < 0.001*** |
| Mean H, μm | 353.57 ± 94.85 | 240.15 ± 69.03 | < 0.001*** |
| Diameter of EZ disruption | 1710.26 ± 488.95 | 1831.62 ± 358.17 | 0.741 |
| Diameter of ELM disruption | 1580.50 ± 468.83 | 1665.72 ± 396.48 | 0.140 |
| DHI | 0.52 ± 0.10 | 0.67 ± 0.21 | 0.013* |
| MHI | 0.28 ± 0.09 | 0.29 ± 0.07 | 0.131 |
| THI | 0.57 ± 0.24 | 0.53 ± 0.42 | 0.689 |
| CME (yes/no) | 21/3 | 5/19 | 0.004** |

*All values are the mean±standard deviation unless otherwise indicated. EZ, ellipsoid zone; BCVA, best-corrected visual acuity; MLD, minimal linear diameter; BD, basal diameter; H, height; DHI, diameter hole index; MHI, macular hole index; THI, traction hole index. P<0.05 is marked with *, P<0.01 is marked with ** and P<0.001 is marked with ***.*
