# Supplementary material for: Predictive value of ellipsoid zone-related angle parameters in primary surgery of large macular hole: a case control study
Source: BMC Ophthalmol. 2023 Nov 17;23:467. doi: 10.1186/s12886-023-03187-7 (PMC10655441; doi:10.1186/s12886-023-03187-7)
Supplement: Supplementary file 3 — Additional file 3: Supplementary Table 3. EZ-related preoperative and postoperative parameter comparisons among unclosed MH patients [file 12886_2023_3187_MOESM3_ESM.docx]

Supplementary Table 3 EZ-related preoperative and postoperative parameter comparisons among unclosed MH patients

| Item | Preop | Post-op | *P* |
| --- | --- | --- | --- |
| Mean EZ-MH angle | 168.35 ± 5.15 | 170.37 ± 3.43 | 0.741 |
| Mean EZ-NFL angle | 78.66 ± 7.49 | 66.61 ± 11.10 | 0.015* |
| AR | 9.31 ± 4.61 | 11.90 ± 5.68 | 0.737 |
| Mean EZ-GCL angle | 75.47 ± 7.77 | 62.07 ± 11.07 | 0.004** |
| AR | 9.20 ± 4.74 | 11.83 ± 6.06 | 0.823 |
| Mean EZ-INL angle | 68.08 ± 8.95 | 45.18 ± 13.58 | < 0.001*** |
| AR | 8.42 ± 4.27 | 11.33 ± 5.36 | 0.117 |
| Mean EZ-OPL angle | 62.91 ± 10.60 | 35.69 ± 14.46 | < 0.001*** |
| AR | 8.84 ± 4.44 | 11.33 ± 4.69 | 0.478 |
| Mean EZ-ONL angle | 59.51 ± 11.63 | 30.02 ± 14.44 | < 0.001*** |
| AR | 8.89 ± 4.35 | 11.34 ± 4.90 | 0.478 |
| Mean Basal angle | 116.11 ± 7.20 | 106.99 ± 13.42 | 0.023* |
| AR | 7.24 ± 4.43 | 14.44 ± 9.30 | 0.005** |
| Mean Basal-NFL angle | 91.53 ± 5.96 | 81.45 ± 10.32 | < 0.001*** |
| AR | 7.95 ± 4.82 | 10.78 ± 6.30 | 0.263 |

*All values are the mean±standard deviation unless otherwise indicated. EZ, ellipsoid zone; AR, angle regularity; NFL, nerve fiber layer; GCL, ganglion cell layer; INL, inner nuclear layer; OPL, outer plexiform layer; ONL, outer nuclear layer; BCVA, best-corrected visual acuity; MLD, minimal linear diameter; BD, basal diameter; H, height; DHI, diameter hole index; MHI, macular hole index; THI, traction hole index. P<0.05 is marked with *, P<0.01 is marked with ** and P<0.001 is marked with ***.*
